# Kelvin–Helmholtz-induced mixing in multi-fluid partially ionized plasmas

**DOI:** 10.1098/rsta.2023.0227

**Published:** 2024-06-09

**Authors:** Ben Snow, Andrew S. Hillier

**Affiliations:** University of Exeter, Exeter, EX4 4QF, UK

**Keywords:** partial ionization, shear instabilities, two-fluid, mixing

## Abstract

Turbulence is a fundamental process that drives mixing and energy redistribution across a wide range of astrophysical systems. For warm ($T\approx 10^{4}\;K$) plasma, the material is partially ionized, consisting of both ionized and neutral species. The interactions between ionized and neutral species are thought to play a key role in heating (or cooling) of partially ionized plasmas. Here, mixing is studied in a two-fluid partially ionized plasma undergoing the shear-driven Kelvin–Helmholtz instability to evaluate the thermal processes within the mixing layer. Two-dimensional numerical simulations are performed using the open-source (PIP) code that solves for a two-fluid plasma consisting of a charge-neutral plasma and multiple excited states of neutral hydrogen. Both collisional and radiative ionization and recombination are included. In the mixing layer, a complex array of ionization and recombination processes occur as the cooler layer joins the hotter layer, and vice versa. In localized areas of the mixing layer, the temperature exceeds the initial temperatures of either layer with heating dominated by collisional recombinations over turbulent dissipation. The mixing layer is in approximate ionization-recombination equilibrium, however the obtained equilibrium is different to the Saha–Boltzmann local thermal equilibrium. The dynamic mixing processes may be important in determining the ionization states, and with that intensities of spectral lines, of observed mixing layers.

This article is part of the theme issue ‘Partially ionized plasma of the solar atmosphere: recent advances and future pathways’.

## 1. Introduction

The lower solar atmosphere acts as a bridge, connecting the low-temperature (≈6000 K) photoshere and the high-temperature ($\approx 10^{6}\;K$) corona. Flux emergence and jets drive material predominantly upwards through the chromosphere, and coronal rain adds material from above, giving rise to a wealth of mixing phenomena that are crucial in determining the energy balance of the solar atmosphere. Turbulent heating occurs as small scales are generated and then dissipated, for example by wave-induced instabilities in prominence threads [1]. Recent studies have also shown that in a mixing layer at the prominence-corona interface, radiative losses become very efficient resulting in cooling that far exceeds the turbulent heating [2,3].

The role of mixing in the heating/cooling of the solar atmosphere is compounded for the warm plasma in the solar chromosphere due to the medium being partially ionized, i.e. consisting of both ionized and neutral species. Partial ionization is thought to play a critical role in heating of the lower solar atmosphere, for example through damping of Alfvén waves [4]. Ionized species are generally considered to be coupled to the magnetic field (i.e. follow the magnetohydrodynamic (MHD) equations), whereas neutral species are generally assumed to not be directly affected by the magnetic field (i.e hydrodynamic). As such, the different species can have different behaviour, for example different waves, different wave frequencies and different criteria for stability [5]. As a simple example, we can look at the linear stability analysis of the Kelvin–Helmholtz Instability (KHI) in an incompressible medium with a sharp jump across the boundary between layers, the stability criteria of a fully ionized fluid is dependent on the strength and orientation of the magnetic field relative to the shear direction, whereas a fully neutral (hydrodynamic) fluid is unconditionally unstable to the KHI, for small perturbations. Thus when combining a plasma and a neutral fluid together to form a partially ionized plasma, the stability criteria of the incompressible KHI will depend on stability conditions for both fluids and the coupling between the ionized and neutral species [6,7]. The same is true for the Rayleigh–Taylor instability in a partially ionized plasma, where the stability and development of small scales are affected by the collisionality [8,9].

In general, partial-ionization is not well understood in the solar atmosphere and can lead to stark departures from fully ionized (or fully neutral) results, for example cooling when single-fluid MHD models predict heating [10]. Previous studies of mixing in partially ionized plasmas have revealed cross-field transport of mass [11] and amplification of the magnetic field strength [12]. However, these models use a simplified form of coupling, with either thermal collisions only, or empirical collisional ionization and recombination rates. Additional effects such as radiative ionization and recombination, and energy loss during ionization/excitation, are essential to physically mimic the mixing and energy balance in solar-like partia ionized plasmas.

In this paper, we study shear-driven mixing in partially ionized plasmas using the comprehensive treatment of ion-neutral interactions presented in [13], which includes both collisional and radiative ionization and recombination for a two-fluid system consisting of a charge-neutral ion+electron plasma, and a hydrodynamic neutral fluid with multiple excited states. In particular, the heating and cooling processes within the mixing layer are studied.

## 2. Methods

### (a) Two-fluid equations

The numerical simulations presented in this paper are performed using the (PIP) code, which solves two-fluid equations for the evolution of a partially ionized hydrogen plasma consisting of a charge-neutral ion+electron plasma (MHD-like) and a neutral fluid (HD-like). The two fluids are coupled through thermal collisions and ionization/recombination [13,14]. The fluids are assumed to consist of hydrogen only. Specifically, the following equations are evolved:

2.1 $$\frac{\partial \rho_{\text{n}}}{\partial t}+\nabla \cdot (\rho_{\text{n}}{\text{v}}_{\text{n}})=\Gamma_{\mathrm{rec}}\rho_{p}-\Gamma_{\mathrm{ion}}\rho_{n},$$

2.2 $$\frac{\partial }{\partial t}(\rho_{\text{n}}{\text{v}}_{\text{n}})+\nabla \cdot (\rho_{\text{n}}{\text{v}}_{\text{n}}{\text{v}}_{\text{n}}+P_{\text{n}}\text{I})=-\alpha_{c}\rho_{\text{n}}\rho_{\text{p}}({\text{v}}_{\text{n}}-{\text{v}}_{\text{p}})+\Gamma_{\mathrm{rec}}\rho_{p}{\text{v}}_{p}-\Gamma_{\mathrm{ion}}\rho_{n}{\text{v}}_{n},$$

2.3 $$\begin{array}{rl} & \frac{\partial e_{\text{n}}}{\partial t}+\nabla \cdot [{\text{v}}_{\text{n}}(e_{\text{n}}+P_{\text{n}})]=-\alpha_{c}\rho_{\text{n}}\rho_{\text{p}}\left[\frac{1}{2}({\text{v}}_{\text{n}}^{2}-{\text{v}}_{\text{p}}^{2})+\frac{1}{\gamma -1}\left(\frac{P_{n}}{\rho_{n}}-\frac{1}{2}\frac{P_{p}}{\rho_{p}}\right)\right]\\ & \;\;\;+\frac{1}{2}(\Gamma_{\mathrm{rec}}\rho_{p}{\text{v}}_{p}^{2}-\Gamma_{\mathrm{ion}}\rho_{n}{\text{v}}_{n}^{2})+\frac{1}{\left(\gamma -1\right)}\left(\frac{1}{2}\Gamma_{\mathrm{rec}}P_{p}-\Gamma_{\mathrm{ion}}P_{n}\right), \end{array}$$

2.4 $$\begin{array}{rl} & e_{\text{n}}=\frac{P_{\text{n}}}{\gamma -1}+\frac{1}{2}\rho_{\text{n}}v_{\text{n}}^{2}, \end{array}$$

2.5 $$\begin{array}{rl} & \frac{\partial \rho_{\text{p}}}{\partial t}+\nabla \cdot (\rho_{\text{p}}{\text{v}}_{\text{p}})=-\Gamma_{\mathrm{rec}}\rho_{p}+\Gamma_{\mathrm{ion}}\rho_{n}, \end{array}$$

2.6 $$\begin{array}{rl} & \frac{\partial }{\partial t}(\rho_{\text{p}}{\text{v}}_{\text{p}})+\nabla \cdot \left(\rho_{\text{p}}{\text{v}}_{\text{p}}{\text{v}}_{\text{p}}+P_{\text{p}}\text{I}-\text{B B}+\frac{{\text{B}}^{2}}{2}\text{I}\right)=\alpha_{c}\rho_{\text{n}}\rho_{\text{p}}({\text{v}}_{\text{n}}-{\text{v}}_{\text{p}})-\Gamma_{\mathrm{rec}}\rho_{p}{\text{v}}_{p}+\Gamma_{\mathrm{ion}}\rho_{n}{\text{v}}_{n}, \end{array}$$

2.7 $$\begin{array}{rl} & \frac{\partial }{\partial t}\left(e_{\text{p}}+\frac{{\text{B}}^{2}}{2}\right)+\nabla \cdot [{\text{v}}_{\text{p}}(e_{\text{p}}+P_{\text{p}})-({\text{v}}_{p}\times \text{B})\times \text{B}]\\ & \;\;=\alpha_{c}\rho_{\text{n}}\rho_{\text{p}}\left[\frac{1}{2}({\text{v}}_{\text{n}}^{2}-{\text{v}}_{\text{p}}^{2})+\frac{1}{\gamma -1}\left(\frac{P_{n}}{\rho_{n}}-\frac{1}{2}\frac{P_{p}}{\rho_{p}}\right)\right]\\ & \;\;-\frac{1}{2}(\Gamma_{\mathrm{rec}}\rho_{p}{\text{v}}_{p}^{2}-\Gamma_{\mathrm{ion}}\rho_{n}{\text{v}}_{n}^{2})-\frac{1}{\left(\gamma -1\right)}\left(\frac{1}{2}\Gamma_{\mathrm{rec}}P_{p}-\Gamma_{\mathrm{ion}}P_{n}\right)-\phi_{I}+\phi_{R}, \end{array}$$

2.8 $$\begin{array}{rl} & \frac{\partial \text{B}}{\partial t}-\nabla \times ({\text{v}}_{\text{p}}\times \text{B})=0, \end{array}$$

2.9 $$\begin{array}{rl} & e_{\text{p}}=\frac{P_{\text{p}}}{\gamma -1}+\frac{1}{2}\rho_{\text{p}}v_{\text{p}}^{2}, \end{array}$$

2.10 $$\begin{array}{rl} & \nabla \cdot \text{B}=0, \end{array}$$

for a charge neutral plasma (subscript p) and neutral (subscript n) species. The fluid properties are given by density ρ, pressure P, velocity v, magnetic field B and thermal+kinetic energy e. Both species follow ideal gas laws for the non-dimensional temperature T, namely $T_{n}=\gamma P_{n}/\rho_{n}$ and $T_{p}=(1/2)\gamma P_{p}/\rho_{p}$, where the specific gas ratio γ=5/3. I is the three-by-three identity matrix.

The species are thermally coupled through the collisional coefficient $\alpha_{c}$, which is calculated as

2.11 $$\alpha_{c}=\alpha_{0}\sqrt{\frac{T_{p}+T_{n}}{2}}\sqrt{\frac{1}{T_{\mathrm{init}}}}.$$

The factor of $\sqrt{1/T_{\mathrm{init}}}$ is to normalize the collisional coefficient using the initial temperature $T_{\mathrm{init}}$ such that $\alpha_{c}(t=0)=\alpha_{0}$.

Ionization and recombination (and excitation, de-excitation) are performed using collisional and radiative rates that are calculated based on the local properties of the plasma, namely the electron number density and temperature. The neutral hydrogen is allowed to exist in five levels (ground + four excited states) and assumed to behave as a single fluid, i.e. the bulk neutral fluid has density $\rho_{n}$, pressure $P_{n}$ and velocity ${\text{v}}_{n}$. The terms $\Gamma_{rec}\rho_{p}$ and $\Gamma_{ion}\rho_{n}$ represent the net recombination and ionization to/from any of the excited state of hydrogen, i.e.

2.12 $$\begin{array}{rl} \Gamma_{\mathrm{rec}}\rho_{p} & \;=\rho_{p}({\hat{C}}_{p,1}+{\hat{C}}_{p,2}+{\hat{C}}_{p,3}+{\hat{C}}_{p,4}+{\hat{C}}_{p,5})/\hat{\Gamma }\\ & \;\;+\rho_{p}({\hat{R}}_{p,1}+{\hat{R}}_{p,2}+{\hat{R}}_{p,3}+{\hat{R}}_{p,4}+{\hat{R}}_{p,5})/\hat{\Gamma } \end{array}$$

2.13 $$\begin{array}{rl} & \;=\rho_{p}({\hat{\Gamma }}_{\mathrm{rec},\mathrm{col}}+{\hat{\Gamma }}_{\mathrm{rec},\mathrm{rec}})/\hat{\Gamma } \end{array}$$

2.14 $$\begin{array}{rl} \Gamma_{\mathrm{ion}}\rho_{n} & \;=(\rho_{n1}{\hat{C}}_{1,p}+\rho_{n2}{\hat{C}}_{2,p}+\rho_{n3}{\hat{C}}_{3,p}+\rho_{n4}{\hat{C}}_{4,p}+\rho_{n5}{\hat{C}}_{5,p})/\hat{\Gamma }\\ & \;\;+(\rho_{n1}{\hat{R}}_{1,p}+\rho_{n2}{\hat{R}}_{2,p}+\rho_{n3}{\hat{R}}_{3,p}+\rho_{n4}{\hat{R}}_{4,p}+\rho_{n5}{\hat{R}}_{5,p})/\hat{\Gamma } \end{array}$$

2.15 $$\begin{array}{rl} & \;=\rho_{n}({\hat{\Gamma }}_{\mathrm{ion},\mathrm{col}}+{\hat{\Gamma }}_{\mathrm{ion},\mathrm{rad}})/\hat{\Gamma }, \end{array}$$

where ${\hat{C}}_{i,j},{\hat{R}}_{i,j}$ represent the dimensional rate coefficients for exchanges from level i to level j. The partial densities $\rho_{ni}$ are the densities of the excited neutral states. $\hat{\Gamma }$ is a normalization factor. ${\hat{\Gamma }}_{\mathrm{ion},\mathrm{col}},{\hat{\Gamma }}_{\mathrm{ion},\mathrm{rad}}$ are the dimensional net ionization rates for collisional (col) and radiative (rad) processes. The excited levels of neutral hydrogen are evolved at each time step using the instantaneous rates.

The heating and cooling terms $\Phi_{R},\Phi_{I}$ are calculated based on the work done on (or by) the free electron during collisional recombination/de-excitation (for heating) and ionization/excitation (for cooling) i.e.:

2.16 $$\Phi_{I}=\frac{1}{\hat{\phi }}(\Sigma n_{i}C_{ip}E_{i}+\Sigma \Sigma n_{l}C_{lu}(E_{u}-E_{l}))$$

and

2.17 $$\Phi_{R}=\frac{1}{\hat{\phi }}(\Sigma n_{p}C_{pi}E_{i}+\Sigma \Sigma n_{u}C_{ul}(E_{u}-E_{l})),$$

where the first term is the energy exchange due to ionization/recombination, and the second term is the energy exchange due to excitation/de-excitation. $\hat{\phi }$ is a normalization factor. The radiative field is assumed to contribute the exact energy required for the transition and thus does not directly contribute to the macroscopic fluid energy. Full details of the ionization/recombination model are available in [13], which is based on the models presented in [15–17].

The radiation rates assume a point-source field with a radiation temperature of $T_{\mathrm{rad}}=6000$ K. An approximation of optically thick rates is applied for the Lyman transitions, where the radiative temperature is set to the local neutral temperature, i.e. the Lyman transitions act as a local blackbody point source.

The model presented in this paper can be compared and contrasted with previous implementations of partial ionization that have been used for studies of the KHI. [2] used the (PIP) code to study the KHI in partially ionized plasmas, where the two fluids were coupled using thermal collisions only (neglecting the effect of ionization and recombination). Other widely used two-fluid models include MPI-AMRVAC [18] and MANCHA [19] that include an empirical form of ionization and recombination. The treatment of ionization/recombination in this paper is significantly more advanced than the commonly used empirical rates and including the such comprehensive treatment of ionization and recombination leads to stark changes in behaviour. An example of this can be found in the modelling of shocks in partially ionized plasma. Using the commonly implemented empirical rates when modelling a partially ionized shock necessitates the reduction of the post-shock temperature [10], whereas the same type of shock using the multi-level treatment results in an increase in post-shock temperature [13]. As such, accurately modelling the ionization/recombination processes can have critical consequences for the heating and cooling process in partially ionized plasmas.

This model used in this paper neglects the role of thermal conduction and visco-resistive terms, which are known to be important in determining the temperature structure and heating profiles. Optically thin radiative losses and heating are included through the $\Phi_{I},\Phi_{R}$ terms; however, this includes hydrogen only. At hotter temperatures ($>10^{4}\;K$), radiative losses from magnesium/calcium resonances and iron become dominant, which are typically modelled using a cooling curve. Only the role of hydrogen transitions are considered in the model used for this paper and the temperatures of interest are below $10^{4}\;K$.

### (b) Energy considerations

In this model, there are a few different categories of energy. The first is macroscopic fluid energy, which comprises thermal and kinetic energy (and magnetic energy for the plasma species). This is the energy directly modelled by the underlying fluid/plasma model given by equations (2.1)–(2.10).

With partial ionization, there is another energy to consider: the energy stored within the ionization/excitation state of the species. The ionization/excitation state energy is not directly modelled by the underlying equations, however can be calculated since the level populations are known at any time instance. Transfer of the ionization/excitation energy to/from the macroscopic fluid via collisional processes leads to energy loss/gain from the plasma fluid due to the work done by/on the electron during these processes. For a collisional-only system with periodic boundaries, the sum of the macroscopic and ionization/excitation energies remains constant through time, i.e. energy remains within the simulation box, but whether it contributes towards the macroscopic energy depends on the ionization/excitation state.

The third type of energy comes from the blackbody radiative field. This is not directly modelled and the total energy in the blackbody field cannot be calculated, however the radiative energy that is interacting with the fluid can be calculated. Radiative processes are assumed to be energy neutral, e.g. during radiative ionization, the photon has the exact amount of energy required to ionize the atom.

### (c) Two-fluid equilibrium with density jump

For an idealized KHI, the pressure should be constant across the interface. However this is not trivial to obtain when the medium is partially ionized with a jump in the density across the interface. A jump in the density with constant total pressure changes the temperature, hence the equilibrium ionization fraction changes. The total pressure also changes since the ion species consists of both electrons and protons, with the proton (or electron) temperature depending on half the plasma pressure. A delicate balance exists whereby the jump in density/temperature across the interface results in no jump in bulk (plasma + neutral) pressure. Specifically, a system is constructed such that the bulk pressure is constant, such that the system should behave like a single bulk MHD fluid as the coupling coefficient tends towards infinity.

The initial conditions are two layers separated at y=0 that feature jumps in density, pressure and $v_{x}$ velocity across the interface, as shown in table 1. Magnetic field is constant and out-of-plane across the entire domain. The system is initially in approximate thermal equilibrium, with the stable density and pressure determined as follows.

**Table 1.** Initial conditions for the simulation. The subscript u (l) denotes the bulk value in the upper (lower) layer. The 2.5D simulation is performed in the x--y plane with the z-direction being invariant. The normalization is such that a density of one corresponds to a number density of $n_{0}=7.5\times 10^{16}\;m^{-3}$. The corresponding temperature of each of the layers in dimensional units is given by $\hat{T}$.

	lower	upper
$\rho_{p}$	1	30
$\rho_{n}$	≈209	≈97
$P_{p}$	1/γ	≈24
$P_{n}$	≈62	≈39
$P_{B}$	≈63	≈63
$v_{x,n;p}$	$v_{l}=(\rho_{u}/(\rho_{u}+\rho_{l}))\sqrt{1/10}\approx 0.119$	$-v_{l}\approx -0.119$
$\text{B}=[B_{x},B_{y},B_{z}]$	[0,0,≈10.95]	[0,0,≈10.95]
$\Gamma_{\mathrm{rec}}$	$10^{-5}$	$\approx 5.5\times 10^{-6}$
$\Gamma_{\mathrm{ion}}$	$\approx 4.5\times 10^{-8}$	$\approx 1.6\times 10^{-6}$
$\hat{T}$	5500 K	7319 K

The first step in calculating an equilibrium with zero pressure gradient is to find the equilibrium on one side of the interface using a specified electron number density and electron temperature and solving the Saha–Boltzmann equation to find the local thermal equilibrium (LTE) state. The Saha–Boltzmann equation is given by

2.18 $${\left[\frac{n_{i}}{n_{p}}\right]}_{\mathrm{Saha}}=n_{e}\frac{g_{i}}{g_{p}}{\left(\frac{2\pi k_{B}{\hat{T}}_{e}m_{e}}{h^{2}}\right)}^{-3/2}\exp\left(-\frac{E_{p}-E_{i}}{k_{B}{\hat{T}}_{e}}\right),$$

where $n_{e}$ is the electron number density (assumed equal to the proton number density, $n_{p}$), $g_{i}=i^{2}$ is the degeneracy of the excitation state, ${\hat{T}}_{e}$ is the electron temperature (in dimensional units), $E_{p}-E_{i}$ is the energy required for a transition between states i and p, $k_{B}$ is Boltzmann’s constant, $m_{e}$ is the electron mass and h is Plank’s constant.

The bulk pressure is defined as $P_{B}=P_{n}+P_{p}=n_{n}T_{n}(1/\gamma )+n_{p}T_{p}(2/\gamma )$, hence the total pressure depends on the ionization state; when a neutral species ionizes it consists of an electron and a proton that both exert pressure, and hence the total pressure increases. The species are assumed to be initially thermally coupled ($T=T_{n}=T_{p}$). The bulk pressure on the lower layer is calculated using a reference electron number density and temperature, which, from the Saha–Boltzman equation, give a neutral number density and hence a total pressure.

A solution is sought that has zero bulk pressure gradient across the interface. To find such a solution, the jump in electron density is specified and a temperature is determined that satisfies the pressure jump equation.

2.19 $$\frac{P^{u}}{P^{l}}=\frac{n_{n}^{u}T^{u}/\gamma +2n_{p}^{u}T^{u}/\gamma }{n_{n}^{l}T^{l}/\gamma +2n_{p}^{l}T^{l}/\gamma }=1,$$

where u,l refer to the properties evaluated in the upper and lower layers.

In this paper, we will use a reference equilibrium using $n_{e}=n_{0}=7.5\times 10^{16}\;{\text{m}}^{-3}$, $T_{e}=5500\;\text{K}$, which give a neutral number density of $n_{n}\approx 208n_{0}$ and thus a neutral fraction of $\xi_{n}\approx 0.995$. We set the jump in the electron number density to 30 and find that the pressure-neutral equilibrium is obtained for $n_{e}=30n_{0}$, $n_{n}\approx 97n_{0}$, $T_{e}=T_{n}\approx 7319$, $\xi_{n}\approx 0.764$. This equilibrium gives physical properties characteristic of thread-thread interactions within prominences. The two layers are connected using a smoothed profile to prevent numerical instabilities caused by discontinuous jumps in finite difference simulations, however comes with the caveat that the thin connecting layer is not in equilibrium at time t=0.

Note that the initial LTE is valid for both the collisional only and the collisional + radiative simulation. The radiative model is defined such that the system reduces to LTE as time tends towards infinity. Further details regarding the radiative model can be found in [13,15,16].

## 3. Numerical simulations

A KHI is driven by specifying a shear flow across the interface. Here, the magnetic field is out-of-plane and thus does not prevent the KHI-instability forming. Boundaries are set to be periodic in the x-direction and zero-gradient in the y-direction. The domain spans x={−0.5,0.5}, y={−0.75,0.75} and is resolved using 1024×1024 cells.

The system is non-dimensionalized using the reference electron number density $n_{0}=7.5\times 10^{16}\;{\text{m}}^{-3}$ such that in the lower layer, the plasma density is $\rho_{p}=1$, and in the upper layer $\rho_{p}=30$. The plasma pressure in the lower layer is set to $P_{p}=1/\gamma$. The bulk pressure is initially constant across the domain at $P_{n}+P_{p}\approx 63$. The plasma-β in a partially ionized plasma can be defined in two different ways. For an isolated plasma, where there are negligible collisions with the neutrals, the plasma-β depends on the plasma properties only and thus plasma-β=0.01. Alternatively, for a fully coupled medium, the plasma-β depends on the bulk properties and thus here bulk plasma-β≈1.44.

The magnetic field direction is out-of-plane such that the magnetic tension does not act to suppress the initial growth of the instability. While the out-of-plane magnetic field prevents magnetic tension and reconnection, magnetic effects arise in the plasma fluid through the magnetic pressure, which confines the plasma and allows sharp structures to form. This is in contrast to the neutral fluid, which is not directly affected by the magnetic field. The initial conditions are given in table 1. The velocity profile has a jump in magnitude of ≈0.24, which when compared with the neutral sound speed in the lower layer of $\sqrt{1/2}$ gives a Mach number of approximately 0.34 implying that compressible effects will increase the stability of the neutrals, but the magnetic field will allow the plasma to develop instabilities more like an incompressible fluid but with magnetic pressure confining the vortices. This difference in behaviour will result in two-fluid effects manifesting. Random noise is specified in the $v_{x}$, $v_{y}$ velocity fields to promote the instability with an amplitude of 0.01.

The collisional coefficient is set to $\alpha_{0}=10$ meaning that collisions occur on simulation time scales of approximately 0.1. The ionization/recombination/excitation/de-excitation rates are normalized such that the collisional recombination rate at time t=0 is $10^{-5}$, i.e. initially recombination occurs on time scales of $10^{5}$ simulation units in the lower domain. It should be noted that the rates are all time-dependent and calculated based on local properties. As the simulation evolves, the rates become enhanced in the mixing layer.

In non-dimensional units, the ion-neutral collision frequency in the lower layer is given by $\nu_{\mathrm{in}}=\rho_{n}\alpha_{c}\approx 2090$. However, in dimensional units, the ion-neutral coupling frequency is defined as

3.1 $$\nu_{\mathrm{in}}=n_{n}\sqrt{\frac{8k_{B}T}{\pi M}}\Sigma_{\mathrm{in}},$$

with the equation of hydrogen $M=1.6735575\times 10^{-27}\;\mathrm{kg}$, Boltzmann constant $k_{B}=1.38064852\times 10^{-23}\;{\text{m}}^{2}\;{\text{kg\textbackslash{}, s}}^{-2}\;{\text{K}}^{-1}$, and the ion-neutral collisional cross-section $\Sigma_{\mathrm{in}}=5\times 10^{-19}\;m^{2}$, which assumes equal temperatures of the ions and neutrals. In the lower domain, this leads to an ion-neutral collisional frequency of $\nu_{\mathrm{in}}\approx 10^{3}\;\mathrm{Hz}$. As such, one-time unit in the simulation corresponds to approximately 2 s.

The underlying numerical code uses finite-difference discretization models and to promote stability, the initially discontinuous jump is spread out over a few grid cells. While both sides of the mixing layer are approximately stable, the material within the smeared discontinuity is not in ionization/recombination equilibrium and thus at very early times, there is ionization/recombination imbalance at the boundary of the mixing layer. This is relatively short-lived and the mixing dynamics rapidly dominate the system.

### (a) Collisional model only (no radiative field)

Firstly, the initial conditions are evolved using collisional rates only, neglecting the role of the radiative field, i.e. the net ionization and recombination are given by

3.2 $$\Gamma_{\mathrm{rec}}\rho_{p}=\rho_{p}({\hat{C}}_{p,1}+{\hat{C}}_{p,2}+{\hat{C}}_{p,3}+{\hat{C}}_{p,4}+{\hat{C}}_{p,5})/\hat{\Gamma }=\frac{\rho_{p}{\hat{\Gamma }}_{\mathrm{rec},\mathrm{col}}}{\hat{\Gamma }}$$

and

**Figure 1.**
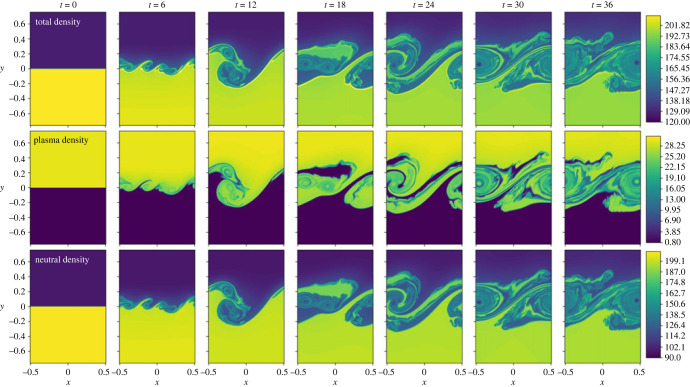
Density evolution for the simulation with collisional ionization/recombination only. Top row shows the total density ($\rho_{p}+\rho_{n}$). Middle row shows the plasma density $\rho_{p}$. Lower row shows the neutral density $\rho_{n}$.

3.3 $$\Gamma_{\mathrm{ion}}\rho_{n}=(\rho_{n1}{\hat{C}}_{1,p}+\rho_{n2}{\hat{C}}_{2,p}+\rho_{n3}{\hat{C}}_{3,p}+\rho_{n4}{\hat{C}}_{4,p}+\rho_{n5}{\hat{C}}_{5,p})/\hat{\Gamma }=\frac{\rho_{n}{\hat{\Gamma }}_{\mathrm{ion},\mathrm{col}}}{\hat{\Gamma }},$$

i.e. only collisional terms are included in the ionization and recombination, with radiative rates set to zero. Note that this model includes the heating and loss terms given by equations (2.16) and (2.17). The purpose of this simulation is to isolate the collisional rate effects, before including the radiative rates in §b. Here, the total energy (macroscopic fluid energy + ionization/excitation energy) remains conserved, however there is conversion between the macroscopic thermal energy of the plasma to the ionization/excitation energy, and vice versa, and thus heating and cooling of the plasma. A time evolution of the density is shown in figure 1.

As the initial conditions evolve, the shearing flow drives the characteristic KHI vortex turnovers that grow and interact, creating a wealth of different scales in the system, with similar structures existing in both the ion and the neutral fluids. The bulk atmosphere is dense in the lower half of the domain, and less dense in the upper half. However, there is a change in ionization fraction across the interface and, whilst the neutrals have the same trend as the bulk fluid (the medium is mostly neutral), the plasma component is the opposite, with the dense region being in the upper half of the domain, see figure 1. For a KHI, the mixing layer is normally extended more into the tenuous material than the dense material, however for the individual fluids here, this would be a different direction. The collisional coupling time scale is relatively short and hence the plasma and neutral couple together well and the general behaviour of the KHI is based on the bulk properties of the medium, and thus the mixing layer extends into the less dense upper region slightly more than the denser lower region of the simulation as predicted in [2].

**Figure 2.**
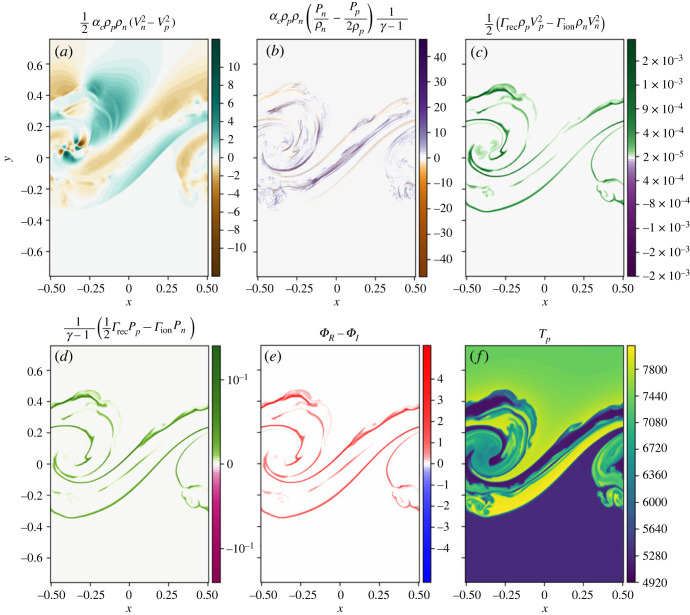
Energy source terms (panels *a*–*e*) and plasma temperature (*f*) for the simulation using collisional ionization/recombination only. Kinetic energy and thermal energy exchange due to thermal collisions (*a*,*b*) and ionization/recombination (*c*,*d*). Panel (*e*) shows the net cooling/heating of the system as a result of the ionization/recombination processes. Panel (*f*) shows the plasma temperature.

The energy exchange terms due to collisions and ionization/recombination are shown in figure 2*a*–*e*. Both of the collisional coupling terms are significantly larger than the ionization/recombination coupling terms demonstrating that the majority of energy exchange between the species is a result of thermal collisions. The energy exchange due to ionization and recombination is predominantly positive, showing that these processes are dominated by recombination. As the hotter and cooler layers mix, ionization and recombination lead to mass exchange between the two fluids. Here, this process is dominated by recombination, i.e. more of the plasma is becoming neutral, than vice versa.

Collisional ionization and recombination (as well as excitation and de-excitation) lead to heating and cooling processes that convert energy from the macroscopic thermal energy of the plasma into ionization/excitation energy that is stored within the ionization/excitation state of the particles and does not directly contribute towards the macroscopic fluid. Here, the recombination is strong in the mixing layer and thus heating bands form at the interface between the layers, as shown in figure 2*e*. Initially, the lower and upper regions of the domain are 5500 K and 7319 K, respectively, however, within the mixing layer temperatures form that are around 8000 K, shown by the yellow contour levels in figure 2*f*. The net heating (due to collisional recombination) in the mixing layer is far greater than the cooling (due to collisional ionization).

**Figure 3.**
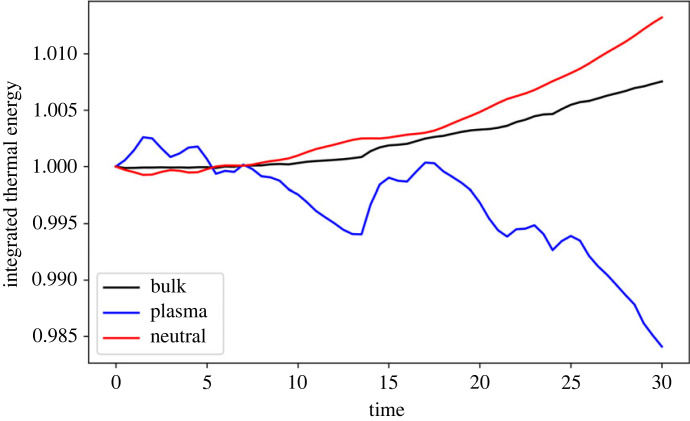
Evolution of total thermal energy through time for the plasma (blue), neutral (red) and bulk (black) fluids for the simulation using collisional rates only. Note each curve is normalized by the respective value of t=0.

Despite the localized heating, the total thermal energy in the simulation box does not change significantly over the simulation time, see figure 3. The temperature of the two fluids is approximately equal, however the thermal energy of each species can change due to changes in the particle numbers. The total (plasma+neutrals) thermal energy increases by ≈0.75% of the value at t=5, however the individual species show vastly different behaviour. The total thermal energy in the neutrals increases, whereas it decreases in the plasma. The medium is becoming more neutral as the simulation evolves due to collisional recombination, which is a heating process of the plasma. However, the plasma and the neutrals are well coupled (through thermal collisions) and thus this energy can be efficiently passed to the neutrals.

**Figure 4.**
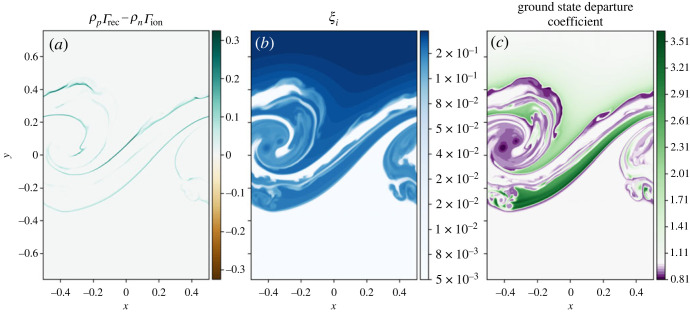
(*a*) Mass exchange due to ionization and recombination, (*b*) ionization fraction ($\xi_{i}=\rho_{p}/(\rho_{p}+\rho_{n})),$ (*c*) departure coefficient of the ground state neutral species.

Figure 4*c* shows the departure coefficient calculated as

3.4 $$D_{i}=\frac{n_{i}}{{\left[n_{i}\right]}_{LTE}},$$

for the level population of neutral state $n_{i}$ from the simulation divided by the LTE level population from the Saha–Boltzmann equation, where i is the excitation state. In this simulation, the level population of ground state hydrogen is significantly greater than any excited level and dominates the dynamics, so only the ground state departure coefficient is shown. Outside of the mixing layer, the departure coefficient is approximately one, as expected, however within the mixing layer the ground state neutral level population differs from the LTE state, even where the system is in approximate ionization/recombination equilibrium, as shown in figure 4*a*. Note that the system is not in true ionization-recombination equilibrium, however the time scales on which the ionization fraction is changing are significantly longer than the dynamic time scales. Neutrals are naturally expelled from the vortex centres (similar to the process seen in gas-dust mixtures [20]) leading to a lower neutral level population and a departure coefficient less than unity, i.e. the neutral level population within the vortex centres is ≈80% of the expected LTE value. There are also large areas where the departure coefficient is greater than unity signifying enhanced neutral level populations compared with LTE. These regions coincide with the enhanced temperature regions within the mixing layer, shown in figure 2*f*. Higher temperature usually results in a higher ionization fraction, however the time scale to reach such an equilibrium is long and thus locally, the neutral population is significantly different than the LTE value.

### (b) Collisional and radiative simulation

Including the radiative field allows for energy to enter or leave the domain and thus can indirectly lead to additional heating and cooling, as the radiative rates can remove or provide ionization/excitation energy that can then collisionally ionize/recombine to alter the macroscopic thermal energy of the medium. Here, the radiative rates are included in the formulations of the net ionization/recombination as given by equations (2.13) and (2.15). The same initial conditions as given by table 1 are evolved, however, here radiation is included in the form of a blackbody radiative field with a radiation temperature set to $T_{\mathrm{rad}}=6000$ K. Lyman transitions are assumed to be optically thick such that the local radiative temperature is given by the local neutral temperature. It should be emphasized that this is an approximation for optical thickness. Further details of the model are available in [13].

**Figure 5.**
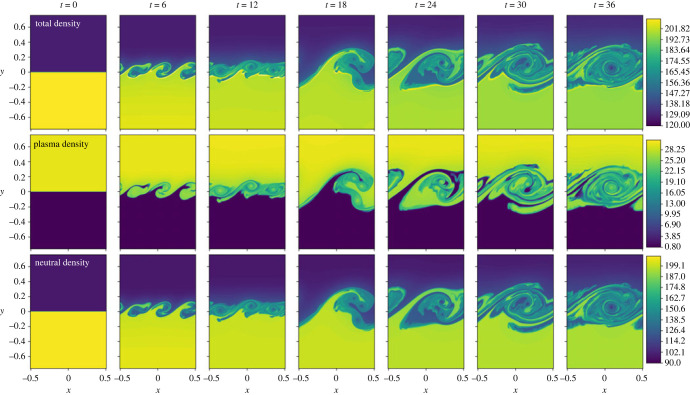
Density evolution for the simulation with both collisional and radiative ionization/recombination. Top row shows the total density ($\rho_{p}+\rho_{n}$). Middle row shows the plasma density $\rho_{p}$. Lower row shows the neutral density $\rho_{n}$.

**Figure 6.**
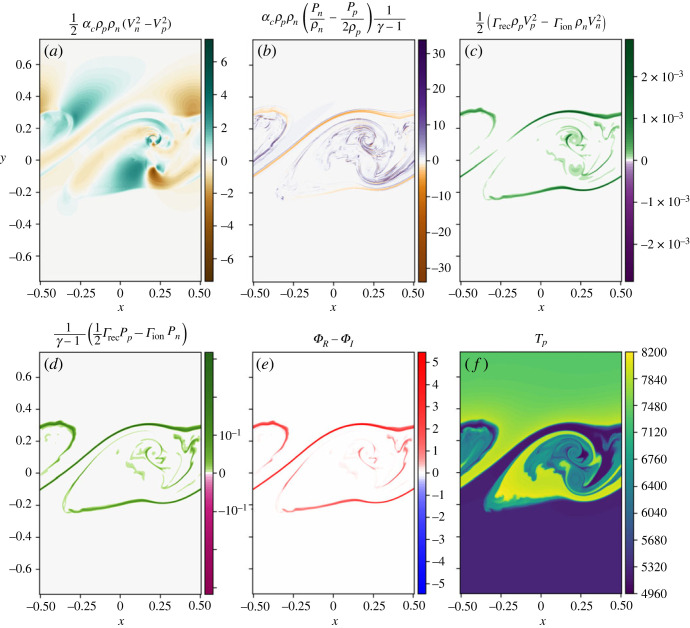
(*a*–*f*) Energy source terms for the simulation with both collisional and radiative ionization/recombination. Here, the rates are the sum of the collisional and radiative rates.

The time evolution of the different densities of the system are shown in figure 5. As before, the coupling is strong enough that similar structures are seen in both the plasma and neutral species. The dominant terms in energy transfer between the two species are again the thermal collisions, see figure 6. The ionization/recombination energy exchange terms are of a similar magnitude to the collisional rates only simulation and are still responsible for less energy exchange between the fluids than the thermal collision terms. The net heating is again always positive within the mixing layer (figure 6*e*), leading to localized heating within the mixing layer, as shown in figure 6*f*.

**Figure 7.**
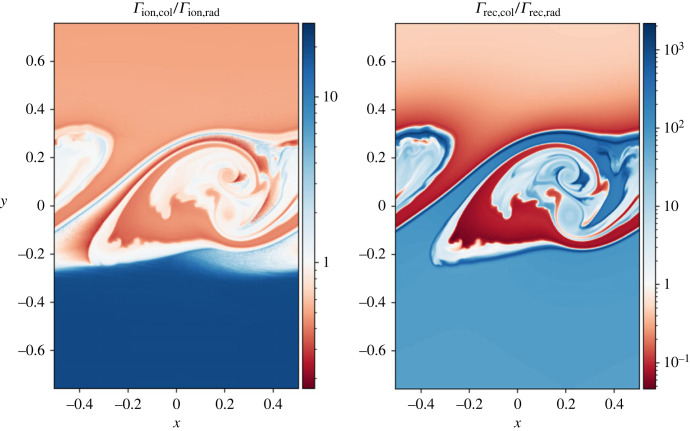
Ratio of collisional and radiative rates at time t=24.

A key difference in this simulation is that the system switches from being dominated by the collisional ionization/recombination rates in the lower domain, to being radiatively dominated in the upper domain, as shown in figure 7. Within the mixing layer, there is no dominant process, with both radiatively and collisionally dominated regions intertwined.

The maximum temperature in the mixing layer is again hotter than the initial temperatures and reaches T≈8200 K, slightly hotter than the simulation with collisional rates only, where the maximum temperature is T≈8100 K. The radiative field does not directly lead to heating or cooling in the model used in this paper, however, the radiative field can indirectly lead to heating through processes such as radiative ionization followed by collisional recombination, where the ionization energy is provided by the photon and work is done on the electron during collisional recombination.

**Figure 8.**
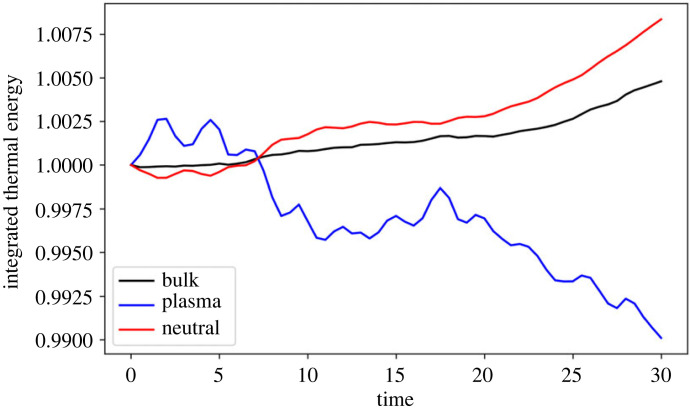
Evolution of the integrated thermal energy in the domain for the simulation with collisional and radiative rates. Note that each curve has been normalized by its value at time t=0.

**Figure 9.**
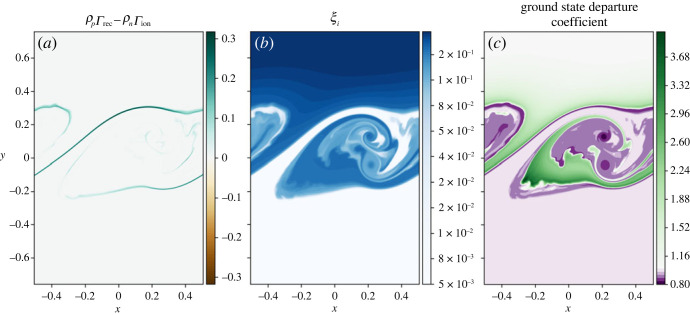
(*a*) Mass exchange due to ionization and recombination, (*b*) ionization fraction ($\xi_{i}=\rho_{p}/(\rho_{p}+\rho_{n})),$ (*c*) departure coefficient of the ground state neutral species.

The integrated thermal energy of the plasma decreases through time, however the bulk (and neutral) thermal energy of the system increases, see figure 8. The relative increases/decreases are comparable with the simulation using collisional rates only. As with the collisional-only model, there is a net recombination near the edge of the mixing layer, figure 9*a*, and a range of ionization fractions due to the mixing of the two layers, figure 9*b*. NLTE conditions exist within the mixing layer, with noticeable depletions of neutral in the vortex centres, figure 9*c*.

## 4. Discussion

### (a) Heating and cooling balance

The KHI instability has been proposed as a heating mechanism, where the small scales generated lead to turbulent heating. However, recent studies have shown that the radiative losses within the prominence-corona mixing layer can result in strong cooling that far exceeds the turbulent heating [3].

**Figure 10.**
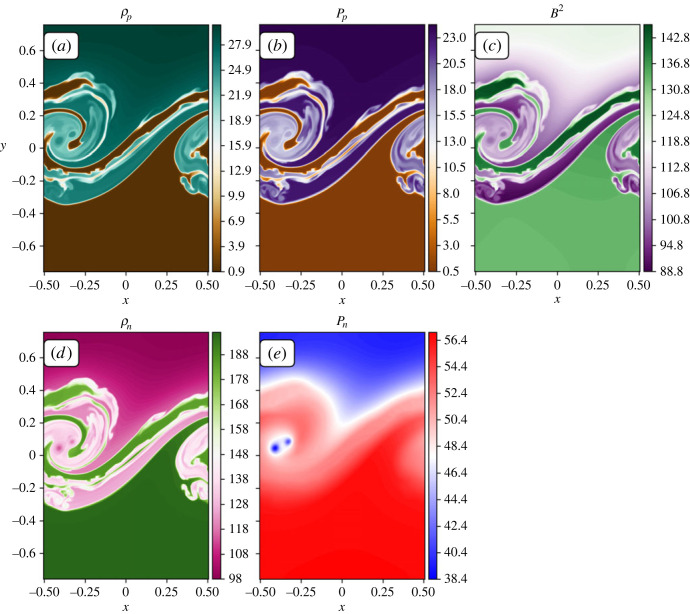
Properties from the collisional rates only simulation at time t=24: (*a*) plasma density, (*b*) plasma pressure, (*c*) magnetic pressure, (*d*) neutral density, (*e*) neutral pressure.

In this work, the mixing occurs between partially ionized regions for a pure hydrogen fluid. Within the mixing layer, both heating and cooling processes occur, with the heating due to collisional recombination being the dominant process. High temperature regions that are hotter than the initial plasma form at the interface between the mixed fluids but the high temperature is not obtained as a result of turbulent heating since these regions are relatively unmixed. This appears to be a result of the pressure distribution in the neutral fluid. In the plasma, sharp structures in the pressure (figure 10*b*) can be supported by relatively small jumps in the magnetic pressure (due to the plasma fluid having a low plasma β), figure 10*c*. In the neutrals, pressure jumps cannot be directly supported (without the development of a shock). As such, the neutral pressure transition layer expands making it far less sharp than the plasma pressure, as shown in figure 10*e*. As new material is brought into the mixing layer, the neutral pressure difference results in regions expanding and contracting to get closer to a neutral pressure equilibrium. A consequence of this is an increase in the neutral pressure in the regions with low neutral density. The combination of a high pressure and low density creates a locally enhanced temperature. The temperature change is quickly coupled to the plasma through thermal collisions and ionization/recombination.

### (b) Dynamical non-local thermal equilibria

The mixing layer in both simulations shows areas that are in an approximate ionization-recombination equilibrium (where the time scales for mass exchange are significantly longer than the dynamic time scales) that is significantly different to the LTE obtained using the Saha–Boltzmann equation, as shown in figures 4*c* and 9*c*. Within the vortex centres, neutral species are expelled leading to a reduction in neutrals compared with the LTE state. Within the hot regions that form, the LTE state predicts far fewer neutrals. In both these regions, the ionization and recombination is occurring slowly and can be considered to be in approximate ionization-recombination equilibrium. Note that the obtained equilibrium is different to the NLTE modelling of prominences that assume a statistical equilibrium, e.g. [21].

## 5. Conclusion

In this paper, shear-driven mixing has been investigated for a partially ionized plasma subject to the KHI. Numerical simulations were performed using the (PIP) code with a multi-level hydrogen model for a case with collisional rates only, and both collisional and radiative rates. The initial layers were set to model mixing between prominence threads, with both the upper and lower regions being partially ionized.

Both the collisional only and the collisional+radiative simulations demonstrate the same overall behaviour where the two fluids are well coupled through thermal collisions, with the ionization and recombination rates being significantly smaller. Within the mixing layer, the collisional recombination rate increases from a background value of $10^{-5}$ to $10^{-2}$ and is responsible for localized heating that leads to a mixed layer that is hotter than either of the initial states. The radiative + collisional simulation heats slightly more than the collisional only simulation. The radiative field provides ionization energy that is then collisionally recombined leading to additional heating. The heating due to collisional recombination/de-excitation in both simulations is far greater than the turbulent heating and thus may be more important in determining the thermal evolution of partially ionized mixing.

In this paper, we have looked at how a new thermodynamic state can be created by mixing, with the mixing layer itself featuring departures from the LTE state. The important implication of this is that dynamical interaction can lead to evolution of the ionization/recombination equilibrium in a prominence or other chromospheric material, and with that the radiative losses of the system. In this special issue, we have a review article that explains the current state-of-the-art in modelling prominence ionization. Our results show that as well as the important NLTE physics created for chromospheric plasma being irradiated by photons from the surrounding atmosphere, an important future step is to understand how dynamics may be driving the change in ionization equilibrium and on the time scales that this can occur in the cool plasma of the solar atmosphere.

## Data Availability

This article has no additional data.
